# Analysis of neurocognitive function and CNS endpoints in the PROTEA trial: darunavir/ritonavir with or without nucleoside analogues

**DOI:** 10.7448/IAS.17.4.19526

**Published:** 2014-11-02

**Authors:** Amanda Clarke, Veronika Johanssen, Jan Gerstoft, Bonaventura Clotet, Diego Ripamonti, Andrew Murungi, Ceyhun Bicer, Maria Blanca Hadacek, Christiane Moecklinghoff

**Affiliations:** 1 HIV and Sexual Health, Brighton and Sussex Medical School, Brighton, UK; 2 Infectious Diseases, Karolinkska University, Sjukhuset, Sweden; 3 Infectious Diseases, Copenhagen University Hospital, Copenhagen, Denmark; 4 irsiCaixa, University Hospital Germans Trias i Pujol, Badalona, Spain; 5 Infectious Diseases, University Hospital, Bergamo, Italy; 6 Janssen, HIV, High Wycombe, UK; 7 Janssen, R&D, Beerse, Belgium; 8 Janssen, EMEA, Issy-les-Moulineux, France; 9 Janssen, EMEA, Neuss, Germany

## Abstract

**Introduction:**

During treatment with protease inhibitor monotherapy, the number of antiretrovirals with therapeutic concentrations in the cerebrospinal fluid (CSF) is lower, compared to standard triple therapy. However, the clinical consequences are unclear.

**Methods:**

A total of 273 patients with HIV RNA <50 copies/mL for over 24 weeks on current antiretrovirals randomized to darunavir/ritonavir (DRV/r) 800/100 mg once-daily, either as monotherapy (*n*=137) or with 2NRTIs (*n*=136). Neurocognitive function was evaluated in all patients by the Hopkins Verbal Learning Tests, the Colour Trail Tests and the Grooved Pegboard Test at screening, baseline and at Week 48. A global neurocognitive score (NPZ-5) was derived by averaging the standardized results of the five domains. In a central nervous system (CNS) sub-study (*n*=70), HIV RNA levels in the CNS were evaluated at baseline and Week 48. Clinical adverse events related to the CNS were collected at each visit.

**Results:**

Patients were 83% male and 88% White, with median age 43 years. There were more patients with nadir CD4 count below 200 cells/µL in the DRV/r monotherapy arm (41/137, 30%) than the triple therapy arm (30/136, 22%). At Week 48, there was no difference between the treatment arms for the five combined domains of the neurocognitive score. At Week 48, the percentage of patients with an abnormal neurocognitive score among the five domains was 12.2% for DRV/r monotherapy and 14.9% for triple therapy. However, one patient on DRV/r monotherapy with a CD4 nadir of 17 cells/µL was hospitalized with HIV encephalomyelitis at Week 24, with HIV RNA 2500 copies/mL in the CSF and 125 copies/mL in the plasma. Symptoms resolved after intensification with high dose zidovudine. A second patient on DRV/r monotherapy with CD4 nadir of 166 cells/µL had a rise in HIV RNA in CSF from <40 copies/mL at baseline to 654 copies/mL at Week 48, with concurrent plasma HIV RNA of 77 copies/mL.

**Conclusions:**

In this study for patients with HIV RNA <50 copies/mL at baseline, there was no difference in neurocognitive function between the treatment arms. However two patients on PI monotherapy with CD4 nadir <200 cells/µL developed viraemia in both CSF and plasma, with one symptomatic case. DRV/r monotherapy should be used with caution in patients with nadir CD4 counts below 200 cells/µL.

